# How do patients actually experience and use art in hospitals? The significance of interaction: a user-oriented experimental case study

**DOI:** 10.1080/17482631.2016.1267343

**Published:** 2017-01-06

**Authors:** Stine L. Nielsen, Lars B. Fich, Kirsten K. Roessler, Michael F. Mullins

**Affiliations:** ^a^ Department of Architecture, Design and Media Technology, University of Aalborg, Copenhagen, Denmark; ^b^ Department of Psychology, University of Southern Denmark, Odense, Denmark

**Keywords:** Health care, healing arts, patient satisfaction, mixed methods, art and interaction

## Abstract

This article aims to understand patient wellbeing and satisfaction and to qualify the current guidelines for the application of art in hospitals. Employing anthropological methods, we focus on the interactional aspects of art in health interventions. A user-oriented study ranked 20 paintings, followed by an experiment using paintings in the dayroom of five medical wards. Fieldwork was done over a two-week period. During the first week, dayrooms were configured without the presence of art and in the second week were configured with the artworks. Semi-structured interviews, observation, participant observation and informal conversation were carried out and were informed by thermal cameras, which monitored the usage, patient occupation and flow in two of the dayrooms. The study shows that art contributes to creating an environment and atmosphere where patients can feel safe, socialize, maintain a connection to the world outside the hospital and support their identity. We conclude that the presence of visual art in hospitals contributes to health outcomes by improving patient satisfaction as an extended form of health care. The article draws attention to further research perspectives and methods associated with the development of art in hospitals.

## Introduction

The tradition of integrating art in hospitals can be traced as far back in history as to when the first hospitals grew out of hostels run by the church for pilgrims and other travellers, and as infirmaries for the sick and the old (Pevsner, 1976). The importance previously attributed to art’s beneficial influence in the hospital environment can be seen in the commissioning of artworks by masters such as Piero della Francesca in Sansepolcro, Hans Memling in Bruges, Matthias Grünewald in Isenheim, El Greco in Toledo, Rembrandt in Amsterdam, William Hogarth in London, Vincent van Gogh in Saint-Rémy-de-Provence and Marc Chagall in Jerusalem (Cork, 2012). In the Middle Ages, art was such a prominent part of a hospital that, while the name of the builder has often been lost, the name of the artist has usually been handed down to our time (Cork, 2012; Stevenson, 1993).

While previously (the art works have now been moved to a museum), Saint John’s Hospital in Bruges in Belgium, for example, had religious images addressing the mind and spirit of patients, today the question regarding the role of art in hospitals is more frequently framed in terms of how art may affect patients’ pain, stress, anxiety and satisfaction within the health care environment. More recent studies of the factors and effects of the physical environments on health outcomes in hospitals have shown a wide range of documented effects on non-clinical and clinical populations, for example of daylight and views to the outside, on blood pressure, stress levels and mortality (Frandsen et al., 2009; O’Bróin, 2015). From these studies, the field of Evidence-Based Design (EBD) has emerged, which holds that the accumulated evidence is enough to conclude that physical design and architecture have effects on both the treatment process and clinical outcomes (Glod et al., 1994; McCaul & Malott, 1984; Ulrich, 1984). This has, in consequence, led to EBD guidelines for art in health care facilities, commissioned with the specific function to contribute to healing (Ulrich, 2009; Ulrich & Gilpin, 2003).

Evidence-Based Design enlists theoretical approaches, such as the Biophilia Hypothesis (Appleton, 1975; Ulrich, 1993; Wilson, 1984), Congruence Theory (Bower, 1981; Niedenthal, Setterlund, & Jones, 1994; Singer & Salovey, 1988) and Distraction Theory (McCaul & Malott, 1984; Miller, Hickman, & Lemasters, 1992; Ulrich, 1984) to justify its claims. While in general these theories are useful in explaining observed responses to the artworks, they are, however, deficient in so far as they fail to address the experience and use of art by the patients themselves in the hospital environment.

The primary question raised by the authors in this study is therefore: How do patients experience and use art in a natural hospital environment, to the extent that they experience or use it at all?

Aligned with the need for, and growth of, qualitative research within art and health care contexts (Crossick & Kaszynska, 2016; Macdonald, 2013), our approach is primarily directed towards the significance of the interactional process, drawing on phenomenological theories of social interaction, spatial interaction and narratives to understand the collected data (Ingold, 2000; Kleinman, 1988; Malinowski, 1922). Within the framework of social and spatial interaction theories, this paper presents anthropological aspects of the experience and use of artworks in a hospital setting, while drawing attention to the relation between art and patients’ degree of satisfaction with the environment.

## Methods

**Figure 1.**
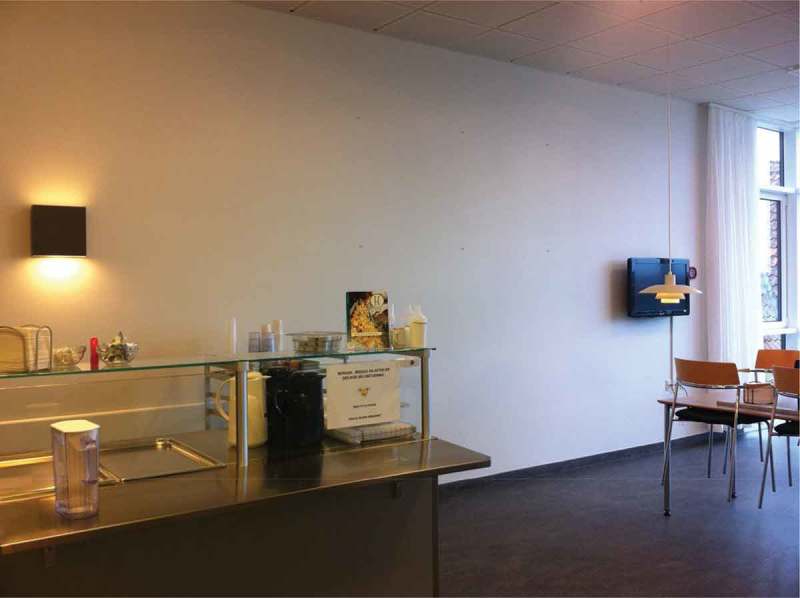
Bare white back wall in patients’ dayroom, Week 1.

**Figure 2.**
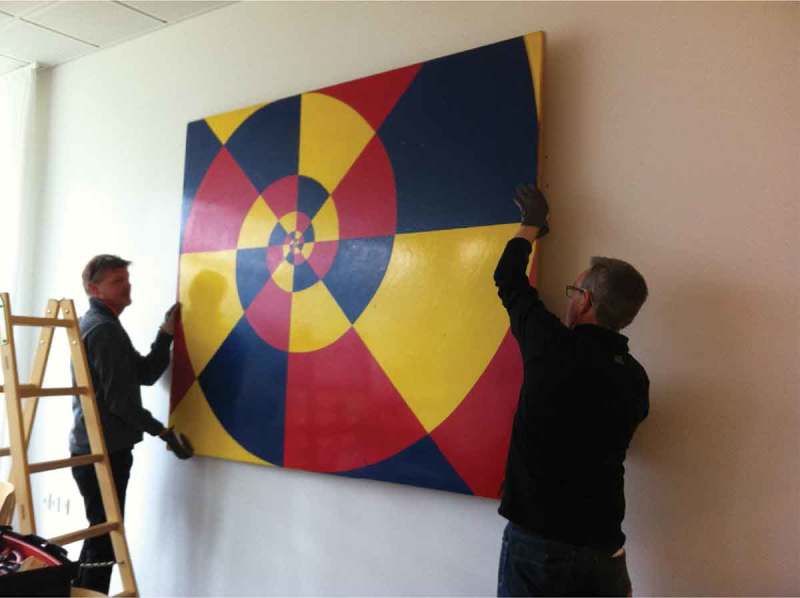
Painting being hung up on back wall in patients’ dayroom, Week 2.

The intervention was organized as natural experiment conducted in the everyday environment of the participants. The experiment was divided into two phases over a two-week period in comparative dayrooms^1^ ^1^The “dayroom” in a Danish hospital ward is typically an open space situated close to, but separate from, the ward and in this case functioned as a place for dining, relaxation, visits, reading, conversation and watching television. of five medical wards at Medicinerhuset, Sygehus Vendsyssel, in Hjørring, Denmark. During the first week (Week 1) all art was stripped from the dayrooms, leaving bare white walls with only spotlights and a television (Figure 1). In Week 2, art pieces were hung up on the back of each dayroom (Figure 2).

### Preliminary ranking study

The art pieces for the experiment were selected based on a preliminary ranking study, conducted over a day in the waiting area for blood sampling at the Copenhagen University Hospital. A total of 103 patients (46% male, 54% female) were given a questionnaire presenting 20 photocopied pictures of artworks and were asked to rank them in relation to which they liked the most, first ranking all pictures from a scale of 1–5 and then ranking their top 5 in relation to each other. The informants’ ages were: under 20 years (1%), 20–40 years (38%), 40–60 years (29%) and over 60 years (32%). They had a short (34%), long (57%) or no (9%) educational background.

**Figure 3.**
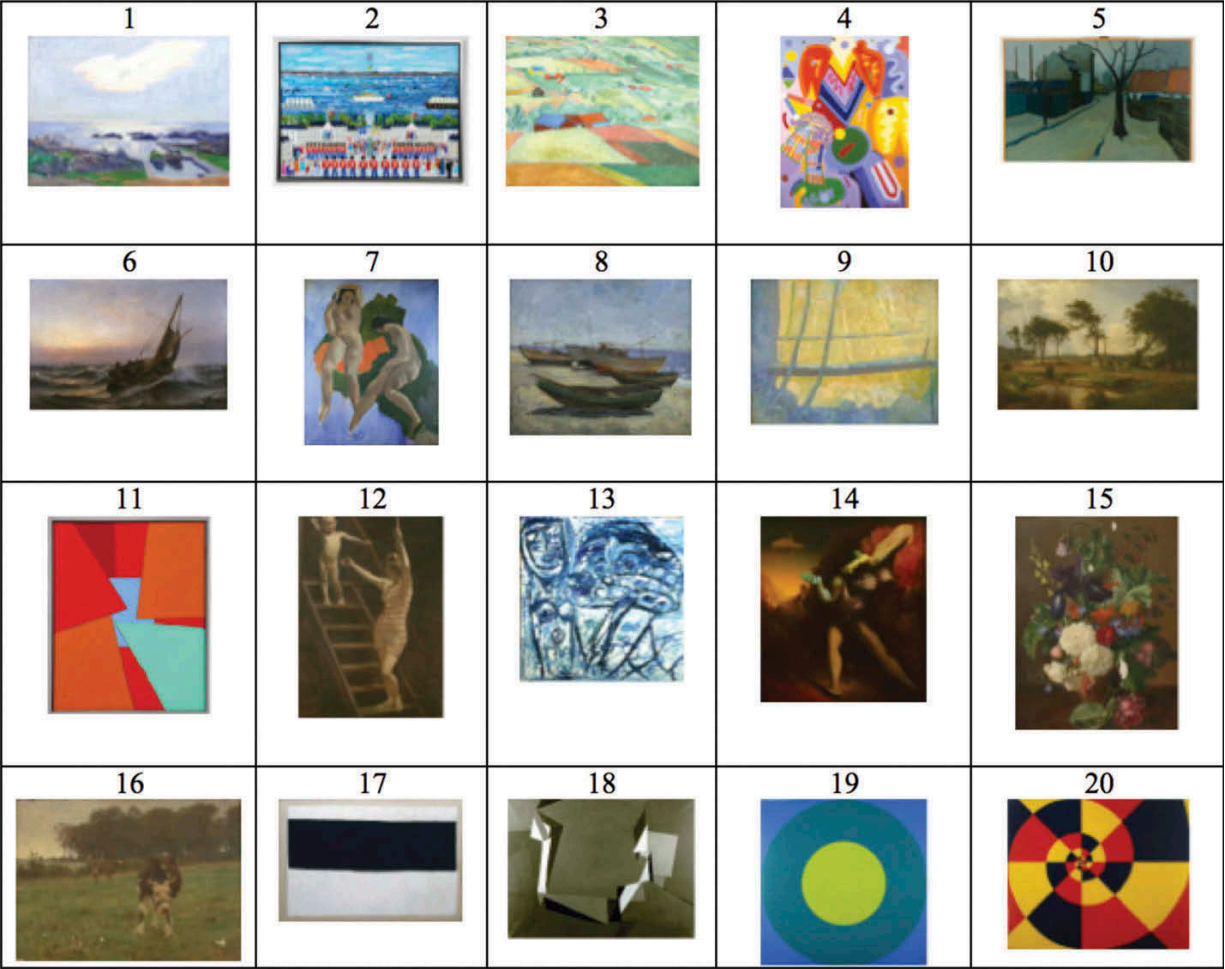
Results of the ranking study (ranking is read horizontally from left to right). *Source*: KUNSTEN - Museum of Modern Art Aalborg.

The 20 art pieces figuring in the ranking study were selected jointly by the first and third author from a pool of 3707 art pieces, from the art catalogue of the Danish art museum KUNSTEN in Jutland, who had offered to lend out five art pieces to the experiment. The authors’ criteria for selecting the artworks were drawn from EBD reports (Diette, Lechtzin, Haponik, Devrotes, & Rubin, 2003; Hathorn & Nanda, 2008; Tse, Ng, Chung, & Wong, 2002; Ulrich & Gilpin, 2003); the intention to have both figurative and abstract in bright and dark colours represented; and a generally open mind. The ranking study thus sought to lower bias and promote data on patient preferences. The patients were presented with a printed copy of 20 pictures (Figure 3).

The four highest and the lowest patient ranked paintings were selected to participate in the experiment in the dayroom of the five wards of the study (art piece no. 4 was not available for loan, and was therefore withdrawn from the experiment). Thus, the art pieces figuring in the case study were nos. 1, 2, 3, 5 and 20.

### Thermal cameras

Thermal camera recording was applied to inform qualitative research with quantitative data on people flow, occupancy and use of the dayrooms, and to supplement the overall qualitative approach and research methods.

**Figure 4.**
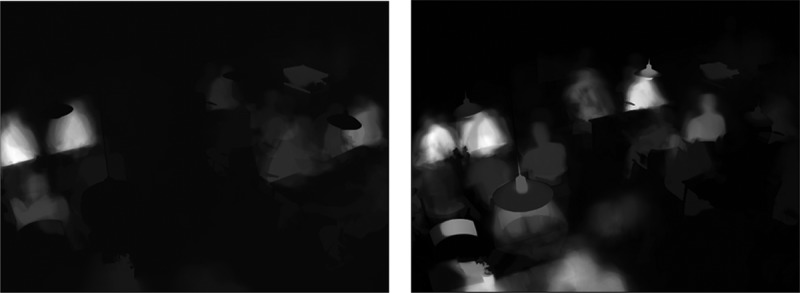
Footage from thermal cameras; dayroom 1—Tuesday, Week 1 (L) and Week 2 (R).

One camera was installed in two of the dayrooms and recorded activity on weekdays between 9 am and 5 pm. The cameras were focused on the back wall of the studied areas, which were white and bare in Week 1 and subsequently decorated with an artwork in Week 2. The cameras only recorded heat radiation from people within the recording frame, with white representing the hottest areas and black the coldest areas of the frame (Figure 4). The cameras did not record identifiable features of the informants; consequently, data from interviews as well as qualitative and quantitative observations are anonymous in accordance with Danish and anthropological ethical guidelines (American Anthropological Association, 2012; Den Landsdækkende Undersøgelse af Patientoplevelser, 2015).

### Interviews and observations

The interviews and observations were collected and designed to accommodate the patient as a departure point and to give an open-ended structure for the coding of interviews, thus minimizing bias (Agar, 1996). We applied 30 semi-structured interviews of a duration time of 8–42 min each (23 min on average). This allowed the participants to reflect and open up for individual experiences, emotions and thoughts (Rubow, 2003; Spradley, 1979). Informal conversations were continuously included, thereby informing the pre-prepared interview guide with concepts met within the field (DeWalt & DeWalt, 2002). Transcripts of interviews and field notes of observations and conversations were subsequently coded using Nvivo software and analyzed individually by qualitative procedures (Madden, 2010; O’Reilly, 2012; Saldana, 2009; Spradley, 1979).

In order to establish the validity of our findings, several steps were taken. When observing the dayrooms, time (date and hour), duration, location (hospital ward no. 1–5) and placement of the fieldworker and participants in the dayroom were consistently recorded for each observation period, in conjunction with outline notes (Emerson, 1995). The analysis strives for transparency of methods, theories, data and empirical settings (Sanjek, 1990).

By applying social mapping methods (Mikkelsen, 1995; Monmonier, 1993), the environment and the practical usage of the dayroom where continuously mapped in Weeks 1 and 2, the internal and external comparison of qualitative data from field observations was facilitated in regards to data collected from thermal cameras.

### Participants

Our focus in the dayrooms was especially on patients’ experience and use of the artworks situated there. We have not taken any further consideration of the diagnostic background of the patients involved in the experiment. However, the participants shared some basic social, cultural and physical characteristics. All participants lived in the northern part of Jutland in Denmark. The 30 interviewed patients were aged 62 on average, ranging from 41 to 91. The participants were predominantly married with children and trained in tradecrafts or agricultural work. All participants where physically mobile and appeared cognitively clear-headed, except for one patient. All observed participants were adult patients ranging from approximately 20–90 years, appearing with the same physical and cognitive states as most of the interviewed patients (Table 1).

**Table 1.** Descriptions of interviewed patients.

Informant	Ward no.^a^	Gender	Age	Place for interview	**Interview duration** (min.sec.)
**Week 1**					
1	1	Male	50	Dayroom	25.21
2	1	Male	54	Patient room	29.55
3	1	Male	62	Dayroom	23.58
4	3	Male	64	Dayroom	19.20
5	1	Male	83	Patient room	32.00
6	1	Female	57	Dayroom	16.54
7	4	Male	65	Patient room	43.17
8	4	Female	46	Patient room	26.01
9	4	Female	50	Patient room	33.40
10	1	Female	61	Patient room	18.09
11	3	Female	63	Patient room	21.03
12	1	Male	52	Patient room	33.44
13	1	Male	53	Dayroom	13.29
14	1	Male	68	Patient room	12.20
15	4	Female	76	Patient room	32.09
**Week 2**					
16	1	Female	63	Dayroom	24.30
17	1	Male	75	Dayroom	16.19
18	4	Male	52	Dayroom	16.34
19	3	Male	64	Dayroom	19.29
20	4	Female	61	Dayroom	27.07
21	1	Male	52	Dayroom	19.47
22	2	Male	91	Dayroom	08.14
23	1	Female	68	Dayroom	21.14
24	1	Female	86	Patient room	25.31
25	1	Male	72	Patient room	42.36
26	1	Male	41	Dayroom	16.57
27	5	Female	53	Dayroom	15.30
28	4	Female	68	Dayroom	24.01
29	3	Male	63	Patient room	30.28
30	1	Female	58	Dayroom	22.35

^a^ Ward and treatment:

1. Cardiac patients

2. Elderly medical patients

3. Lung patients

4. Gastro patients

5. General internal medicine.

### Topics mentioned by the patients

The patients talked about several different themes during the informal conversations and semi-structured interviews. As illustrated in Table 2, every theme was detected by the Nvivo-coding of the semi-structured interviews and categorized into more general topics from a iterative-inductive research approach (O’Reilly, 2012). To ensure intercoder reliability, themes in the qualitative data were guided by themes from earlier qualitative studies on patients’ wellbeing in relation to physical surroundings in health care settings (Nielsen, 2013; Timmermann, 2014), studies on art in hospitals (Nanda, Eisen, Zadeh, & Owen, 2011; Ulrich & Gilpin, 2003) and methods for conceptualisation and perception of art (Arnheim, 1954; De Botton & Armstrong, 2013) in the coding process.

**Table 2.** Example of the coding of an interview.

Patient quotes (internals)	Recurrent themes (nodes)	Overall topics (memos)
I like it when there is a sort of play in the surroundings, for example when working with wood…you see the years of the tree and sense the nature…it’s alive. Thus, something happens when surfaces are three-dimensional and moving. Here it’s different with this the all-white room…you may as well stay in a snow cave—here is nothing to relate to and it feels very cold.	Sensory stimuli Texture	Environment and interaction
	Nature Time Life	Nature and life
	Shape Depth Movement	Art and motive
	Colour	White vs. colour
	Identity Sensation	Dwelling Atmosphere

In total, the processing of data detected 63 recurrent themes such as *time*, *safety, nature*, *movement*, *colour* and *memories*, which were categorized into 21 overall topics—all relating to the patient experiences and uses of art in hospitals.

## Analysis

Observation, to be of value, must lead to insight, the noticing of apparently insignificant points, the making of connections, the discovery of what Henry James (1962) called “the figure in the carpet.” (Cohen, 1984, p. 220)

The analysis and the topics presented in this paper have been chosen by their frequency of occurrence in the dataset, their inter-correlations and their ability to inform evidence and awareness of the potential of art in hospitals. The following analysis thus combines the overall topics detected in the dataset towards a phenomenological understanding of the experience and usage of art by hospitalized patients.

The following two overarching themes, detected by the coding of the qualitative and quantitative case study data, indicate the significance of art in hospitals from a perspective of patient satisfaction:

- Art and expectation
- Art and interaction

These themes are used to structure the analysis. The first theme, “Art and expectations,” serves as the initial departure point, in relation to the overall contextual role of art in relation to hospitalized patients. The second theme, “Art and interaction”, goes in depth into how art was experienced and used by patients during their hospital stay in four stages of effect; Physically, Socially, Emotionally and Cognitively.

### Art and expectation

When interviewed about their experiences of their hospital sojourn, the majority of the patients expressed an overall positive feeling of approachability and care, where both nurses and doctors were described as competent, informative and welcoming. Several patients did not notice the bare wall in Week 1, nor the artwork installed in Week 2. It was primarily when first asked about the physical surroundings that they expressed these as meaningful and a subject for their attention (Table 3).

**Table 3.** Patient quotes on the theme “art and expectation.”

*A: “I feel comfortable here… I am treated really well” I: Do you think the rooms have something to say in this? P: No, I don’t think so… but hadthe staff not been nice and friendly, then the room would have been of greater importance… but with the tranquillity and warmth radiating from the staff it outweighs the surroundings.”*
(Interview, week 1)
*B: “I don’t think it could be any different in a hospital (than these bare walls)”, “… I’m not in need for more (décor)”and “Well, I would regard pictures or paintings and things so luxurious to hang up in here… I’m only going to be here for a short period of time you know.”*
(Interview, week 1)

The weighting of the social environment (concerning human contact and medical treatment) over the hospital’s physical environment, its décor and art appeared as a continuous theme (Table 3, A). Hereto, the study’s participants generally expressed little or no expectations to the role of art in the hospital (Table 3, B).

However, as the analysis “Art and interaction” will show, this did not in itself rule out art as a significant generator of patient satisfaction in hospitals. Towards approaching the phenomenon of the patients’ lack of expectations of the physical environment in hospitals, we apply a framework of *narrative theory*, which includes aspects of sociocultural context (Good, 1994; Kleinman, 1988; Mattingly, 1998).

In the annual Danish nationwide survey of patients’ experience and satisfaction of hospitals (Den Landsdækkende Undersøgelse af Patientoplevelser, 2015), no questions directly address the role of the physical environment on patient satisfaction. From a contextualizing point of view, the political and cultural context of patients is given little or no room for their comment or reflection. The survey reflects a sociocultural context in which the medical paradigm predominates as a measure of patient satisfaction, and in which patients’ expectations to the physical environment are secondary, in relation to successful health outcomes. As one participant expressed it, when asked what he expected of the hospital and his stay there: “To get well! And then get back home again.” The lack of attention to the presence of art in hospitals is a matter of priority and focus, by the patients themselves as well as the medical-care system, rather than their expression of art as meaningless or unimportant in that environment.

In theoretical terms, patients’ creation of meaning in their experience of hospital stay can be seen as the result of a process of interpreting their impressions on the basis of a “plot,” generated and guided by their social and cultural context (Bruner, 1986; Good, 1994). In relation to their experience of the hospital, the patients’ narratives prioritize medical care over aesthetics. In other words, patients filter impressions received from the hospital environment through a narrative, derived from their sociocultural contexts.

Where the social and cultural context of patients thus becomes a central element in the understanding of how art is experienced in hospitals, an application of narrative theory suggests that an apparent lack of awareness of the artworks should not be interpreted as an expression of its non-effect. Consequently, to investigate the potentials and limitations of art as an integrated part of patient satisfaction in hospitals, we stress the need to take a larger social and cultural context into consideration when interpreting data.

### Art and interaction

The second of primary themes detected in our data set is the potential of art to interact with patients. The modes of interaction are structured for this initial and overall analysis into four stages of effect: physical, social, emotional and cognitive. Thus, this analysis of data prioritizes the qualitative findings on patients’ experiences and usages of art in Week 2 of the experiment, rather than correlating experimental findings in changes from Week 1 to Week 2.

#### Physical effects: art that moves bodies

When interviewed about where they chose to sit or stand (their placement) in the observed dayrooms, few patients expressed a conscious choice of placement in relation to the artworks, but gave other motivating factors. These factors, which are identified from the interviews, field observations and thermal camera footage, can be divided into two categories: (1) psychosocial factors (socialisation vs. privacy, habit, ease and unease), (2) spatial factors (access to daylight, view, room for manoeuvre, accessibility).

The primary psychosocial parameter for patients’ placement in the dayrooms was whether they wished to be private or to socialize with other patients or relatives; that is to say, whether they chose to sit alone or together with others. The primary spatial parameter for placement was access to daylight and a view to the world outside. However, whether sitting alone or with other people, the patients in both cases preferred to sit by the window.

In an additional appraisal of psychosocial and spatial factors in placement, the qualitative and quantitative data indicated a tendency for patients to sit or stand with their backs against the back wall of the dayroom. This occurred more frequently when the wall was covered with art (Week 2) than when it was bare and white (Week 1); thermal cameras detected more heat radiation near the end wall in Week 2 than in Week 1 (Figure 4) and notes from field observations describe the same tendency (Table 4, C)

**Table 4.** Notes and patient quotes on the theme “art and interaction.”

**Physical**	
*C.*	*A patient is sitting alone in the empty dayroom with his back to the Gernes-painting (no. 20). Another patient comes into the dayroom and asks if she can sit down next to her. Both patients are now sitting with their backs to the painting. A third patient comes and sits opposite the two (facing the painting). After a while of silence they speak of herring and soup over lunch*. (Observation note, week 2)
***Social***	
*D.*	P1: *You have a good feeling out here… if only there were more people…* P2: *I never experience many people out here… they are probably all too sick… they lie in their beds and get food served in there. There is rarely a big get-together out here*. (Informal conversation note, week 1)
*E.*	*It makes a difference that the walls are covered with art… otherwise it's just all white and un-cosy. It puts you in a more comfortable mood when you are hospitalized. It’s like there is something to look at… and something to build on. Something you can sit and talk about and take home with you…* (Interview, week 2)
*F.*	P1: *Have you seen the picture that has been hung up? Hmm, it’s a bit boring… maybe it's not quite finished*. P2: *It isn’t something I would have hanging on my walls at home… P3: No, it's maybe a little too similar in colour… But they are fresh…* (E-F: Interviews, week 2)
**Emotional**	
*G.*	*They (the staff) can’t run around and switch paintings each and every other day… it can’t be done… but it's great with just a little decoration on the walls… to know that the surroundings are also taken into account*. (Informal conversation note, week 1)
*H.*	*I sense the space more defined… not as a place where I am free to observe.*
*I.*	*I like this painting. It gives me an urge to sit down and relax… Like in a cage.*
*J.*	*… (The art) provides safety… you feel yourself shielded in a way.* (H-J: Interviews, week 2)
*K.*	*I think I would be more relaxed (if there had been something on the wall), instead of this white wall… if there had been something on the wall the room had probably not been (felt) so large, I think…*
*L.*	*(…) The room only holds some cafeteria tables… it's a bit like a canteen in a workplace. The white wall over there might also be a little nicer if it were decorated… Such a wall looks so bare.*
*M.*	*It’s different with this all-white room… you may as well stay in a snow cave - here is nothing to relate to and it feels very cold.* (E-F: Interviews, week 1)
*N.*	*It’s a funny one that one (painting)… I look at it every day… one always finds something new. I noticed… the small people in the middle and behind the statue… And then there are also the whales on the right side of the boat over there. There once was a boat sailing from Copenhagen to Aalborg… you can easily make some stories out of this painting… in my younger days we caught the big tunas in Øresund that pulled up to spawn in the Baltic Sea… I fished for 10 years you know…*
*O.*	*I like this painting a lot… I look at it all the time… I just feel it's my painting you know… it’s Vendsyssel, it’s the landscape I have grown up in and it's the storm and the winds I have lived in… (…) One doesn’t expect much of the decoration in a hospital… I once was hospitalized in a another hospital, where the walls where all white… then there was only one's own life and the lives of your children to think about… it's not a good thing just to have that. I: What if the painting had not been here? P: Hmm… it’s as if I'm living in a different world now you know… all I think about is my boys… therefore it (the art) may not be so crucial right now… I lie in bed and don’t have any powers left… it's a bit as if you step into yourself in a different way… I do this you know… in that way it appears secondary to me (…) I: But it can be rewarding to have something looking at? P: Yes, absolutely… last night I sat here on a chair when a flock of birds came flying by… things like that, they infuse something… some sort of life… you have a need for that lying in a place like this…”. “* (N-O: Interviews, week 2)
**Cognitive**	
*P.*	*It is great to have something else to look at, when you have finished counting all the dots in the ceiling… when you’ve been here for a week, you can easily get a sort of cabin fever…*
*Q.*	*It looks like a garden snail .. Or maybe something with a projectile… One can’t help looking at the center. You get sort of focused. It's more quiet than uneasily… You just wait for it to continue*. (P-Q: Interviews, week 2)

While the patients apparently weren’t aware of the artwork as an important element of their bodily praxis in the environment when asked, their movements however indicated signs of their tacit awareness of the artwork’s presence, presumably as something infusing more ease and safety than having their back up against a white wall or looking at the art. As anthropologist Daniel Miller writes: “The surprising conclusion is that objects are important, not because they are evident and physically constrain or enable, but quite the opposite. It is often precisely because we do not see them” (Miller, 2010, p. 50).

#### Social effects: art as a three-dimensional socializer

The case study indicates that artworks influence the practice and atmosphere of sociability.

Data from field observations and statements from the semi-structured interviews show how patients were inclined to use the dayroom in relation to other people. Footage from the thermal cameras supports this finding (Figure 4). Most of the participants were physically mobile; and while this is not possible for more immobile patients, their use of the dayroom was often motivated by the introduction of variation into their daily lives and a break from medical treatment.

Most of the case study participants wished for a fuller social environment in the hospital (Table 4, D). Once again, their statements showed how the social environment of the hospital ward is prioritized in the patients’ experience and how this influenced their feelings of satisfaction toward the experience.

More particularly, the artworks showed a positive effect on the social environment in the dayroom by activating patients to engage in dialogue (Table 4, E). Hereto, collation of field observations and interview data from Weeks 1 and 2 shows how patients in Week 2 had a higher frequency of socializing with other patients, relatives and staff, experiencing and using the artwork as a common frame of reference (Table 4, F).

This form of phatic communication established and maintained interaction as a way to infuse an “atmosphere of sociability” (Malinowski, 1922, pp. 313–315). Thus, the artworks in the present case promoted socialisation and atmosphere, engendering a mood of togetherness, ease and relatedness.

The study of art’s potential in hospitals thus reveals three new dimensions of how art can serve as a positive distractor, in praxis, speech and atmosphere, thus adding depth to prior studies that have primarily focused on the psychological effects of art (Glod et al., 1994; McCaul & Malott, 1984; Ulrich, 1984). This three-dimensional view of art as distractor through praxis, speech and atmosphere influences patient satisfaction by interacting and colouring their experience and mood.

These findings do not entirely accord with the theory of *emotional congruence*, which posit that current emotional states will bias patients’ responses to environmental stimuli (Bower, 1981; Singer & Salovey, 1988). This approach is frequently employed in environmental psychology studies that have examined patients’ experiences of art (Hathorn & Nanda, 2008; Ulrich, 2009; Ulrich & Gilpin, 2003) and that have led to discouraging the use of abstract art and to encouraging easily recognizable figurative motifs. Our findings, however, indicate that patients’ perceptions may be formed in their interaction with artworks. The emotional state of the patient can be positively affected, regardless of the artwork’s degree of abstraction, by its potential to generate sociality and atmosphere. There is, however, a need for further investigation into art as an agent, interlocutor and a generator of atmosphere, which lies beyond the scope of this case study.

#### Emotional effects: art upholding security, safety, self and spirit

The data shows how art interacts with patients on an emotional level, promoting composure in different ways, such as an expression of unexpected luxury, a definer of space and a generator of memories. In relation to low expectations to the art décor in hospitals and the notion of art as a luxury, patients expressed an experience of art as surplus (Table 4, G).

The quotations exemplify how patients generally experienced the presence of art as a comforting additional element that went beyond direct medical treatment and care. The underlying notion appears to be that if the hospital has resources and energy to consider the physical environment, then the fundamental medical responsibility of the hospital must be in order. In this case it is not just the fact that art as material is present, but also the fact that it represents a greater context that leads patients to experience a sense of security.

Furthermore, interviewed patients expressed how they found it easier to navigate in the dayroom in Week 2 and that this made them feel safer and more protected. This could in part explain the higher number of interviews in dayrooms in Week 2, compared to the number of interviews in patient rooms in Week 1 (Table 1) and the fact that patients expressed an overall experience of satisfaction in Week 2 (Table 4, H–J). In contrast, the experience of safety, shielding and relaxation in the dayrooms were not expressed in Week 1 of the study. Here, patients experienced to a greater extent the dayrooms as cold, big, institutional, uninspiring and uncomfortable (Table 4, K–M).

The differences in patient experiences between Weeks 1 and 2 thus indicate sensations of spatial tranquillity and serenity in the presence of artworks. From a phenomenological approach to the human bodily sensation and experience of space (Galvin & Todres, 2011; Ingold, 2000), these sensations express firstly, how art reduced perceptions of spaciousness, underpinning psychological environmental studies, which argue that white rooms are experienced as more spacious than coloured rooms (Kwallek, 1996), and secondly that art had the potential to instil a sense of ‘dwelling’, ‘at homeness’ and well-being to a higher degree than a hospital space without art. In the third place, the artworks affected composure and patients’ emotional states through the generation of memories, which stimulated, activated and addressed the majority of patients on a personal level (Table 4, E, N–O).

Patients experienced that art had the potential to positively distract them from uncomfortable situations of illness, by directly addressing their memories of themselves as individual, social and cultural beings. This affect not only showed to be of great significance for the physically mobile and relatively well-recovered patients, but was also important for the weaker and dying patients. In the latter cases, one art piece in a patient room (not part of the case study art) provided existential meaning by generating memories and fulfilling spiritual aspirations for one terminally ill patient (Table 4, O).

Furthermore, for some patients, art becomes a tool for existential support and a connection to life. From his studies on elderly people, the Norwegian sociologist Lars Tornstam (1997) developed the concept of *gerotranscendence*: “Simply put, gerotranscendence is a shift in meta-perspectives, from a materialistic and pragmatic view of the world to a more cosmic and transcendent one” (p. 1). The idea that elderly people, at least at times, are more prone to sense the world in a certain meditative state of contemplation, correlates with the experience of the dying patient quoted above. The potential of the artwork in this particular state of mind is as a generator of memories, through which life experiences can be assembled into a meaningful whole.

The study contributes to current evidence on art as a positive distractor and its potential as an easer of pain, anxiety, fatigue and stress (Diette et al., 2003; Frandsen et al., 2009; Gershon, Zimand, Lemos, Rothbaum, & Hodges, 2003; Tommaso, Sardaro, & Livrea, 2008; Ulrich, 2008; Ulrich & Zimring, 2008). The study shows that art has the potential to positively distract patients by addressing them socially, culturally and existentially as individual human beings. As medical anthropologist Kleinman (1988) has argued through his dichotomy of illness vs. disease, these effects are particularly important in relation to patients who experience a deprivation of identity and power during hospitalization.

#### Cognitive effects: art and the notion of time, life and movement

The political agenda in Denmark has in recent years focused on reducing waiting time in hospitals (Center for Kvalitetsudvikling, 2008). The reduction of waiting times and unplanned delays for those who receive, as well as those who give, health care is indeed a constant topic and focus in the health care industry (Henriksen, Isaacson, Sadler, & Zimring, 2007).

In general, the patients interviewed did not experience undue waiting times, but the experience of time passing slowly and feelings of restlessness were often expressed (Table 4, P). Comparing interview data from Week 1 and Week 2 reveals that patients appear to experience “slowed time” less in Week 2 than in Week 1. While patients commonly experienced the white wall as less inspiring and harder to relate to, the general experience in Week 2 was of calmness, memory, togetherness and derestriction. Thus, spatial stimulation, the positive effect of the environment on mood, is higher in Week 2 than Week 1. When comparing data from informal conversations, interviews and field observations, patients seemed to prefer surroundings where “something is happening” and where “the surface is broken,” as also stated in the patient quote of Table 2 and quote Q of Table 4.

Life and movement in hospital surroundings has been studied previously in a social and physical perspective in terms of wayfinding and human relations (Frandsen et al., 2009, pp. 113–124). It is, in Denmark at least, a commonly held view that experience of healthcare facilities is improved through art and that art in general has a potential to “infuse life” into its physical surroundings (Hessov, 2005; Simonsen et al., 2012). However, empirical studies on art and movement—both movement in art and movement generated by art—have been studied to a lesser extent (Glod et al., 1994) and there is limited knowledge on if, how, why and what kind of art has this influence.

This enquiry does indicate that certain types of artworks facilitate a more positive aesthetic reaction than others among the patients interviewed. An explanation for this can be sought in *fluency theory* of aesthetic pleasure, which concerns how people experience beauty and which suggests that aesthetic experience is a function of the perceiver’s processing dynamics. Hence, the more fluently a perceiver processes an object like an artwork, the more positive is their aesthetic reaction (Reber, Schwarz, & Winkielman, 2004). To what extent fluency theory may be used to explain these aspects of the data and observations, together with which types of artworks underpin positive aesthetic reactions among hospitalized patients, requires further study.

## Discussion

Data were collected through mixed methods, including 30 semi-structured interviews with hospitalized patients, thermal camera surveillance and two weeks of fieldwork in five medical wards. However, the research methods and design applied have been primarily qualitative, complemented by quantitative analysis of recordings by thermal cameras where the qualitative analysis was unclear. For the further development of combining these methods, and the collection of statistically significant data, we recommend camera recordings over a longer period of time and in contexts with a higher flow and use of space.

The patients interviewed were all users of the dayroom area and were generally admitted for short periods of time; in consequence, the immediate experience and use of the artworks is by relatively “fresh” patients. While this reflects the limited focus of the study on a certain type of patient within the complex environment of the hospital, it gave the opportunity for collecting interviews with different patients and personalities over a short period, leading to the collection of larger amounts of observations and data.

The natural experiment design employed includes the danger of confounding and uncontrollable factors; however, the patient behaviour studied reflects a very high ecological validity, as it occurs in non-laboratory, true-to-life settings.

Studies are still needed on the potential effects of art on seriously ill patients hospitalized over a longer period and in settings other than the dayroom. These studies will need to be designed with the illness of patients in mind, where their level of frailty requires more sensitive interventions.

## Conclusion

Studies in the effects of visual art on health outcomes have generally employed theories drawn from the fields of psychology and biology. This article approaches the question of the effects of artworks on patient satisfaction primarily from an anthropological point of view.

The question initially raised by the study was “How do patients experience and use art in a natural hospital environment to the extend that they experience or use it at all?” It has been found that art has the potential to positively affect patients’ satisfaction with their sojourn in the hospitals studied. Although visual art occupies the background in patients’ experience of hospitals, it influences patients’ experience of safety, comfort, time and identity.

The study generally brings a greater understanding to how art is experienced and used in the daily life of hospitalized patients: their experience, their use of space in hospitals and ultimately that art affects the degree of satisfaction with hospital treatment. The application of anthropological perspectives is useful in providing new perspectives in the exploration of art as experience and effect in health care settings.

Focusing on the social and cultural effects of artworks in hospitals, the study verifies aspects of previous evidence-based studies of visual art in hospitals, but indicates the need to qualify and re-examine the theoretical perspectives widely employed in the field. While the analysis raises questions about the currently available guidelines for art in hospitals, particularly regarding the potential of abstract art to have positive effects, the study confirms that art promotes an experience of enhanced quality and satisfaction among patients. The artworks contribute in creating an environment and atmosphere where patients can socialize and stay connected to the world and life outside the hospital, themselves and their spirit. The experimental case study thus indicates that visual art contributes positively to health outcomes in hospitals.
